# Effectiveness of Web-Based Self-Disclosure Peer-to-Peer Support for Weight Loss: Randomized Controlled Trial

**DOI:** 10.2196/jmir.2405

**Published:** 2013-07-09

**Authors:** Mie Imanaka, Masahiko Ando, Tetsuhisa Kitamura, Takashi Kawamura

**Affiliations:** ^1^Health ServiceKyoto UniversityKyotoJapan; ^2^Center for Advanced Medicine and Clinical ResearchHospitalNagoya UniversityNagoyaJapan; ^3^Division of Environmental Medicine and Population SciencesDepartment of Social and Environmental MedicineOsaka University Graduate School of MedicineOsakaJapan

**Keywords:** obesity, weight loss, health support, self-disclosure, email, randomized controlled trial

## Abstract

**Background:**

Obesity is one of the most common public health problems in the industrialized world as a cause of noncommunicable diseases. Although primarily used for one-on-one communication, email is available for uninterrupted support for weight loss, but little is known about the effects of dietitian group counseling for weight control via the Internet.

**Objective:**

We developed a Web-based self-disclosure health support (WSHS) system for weight loss. This study aims to compare the effect of weight change between those using the WSHS and those using the email health support (EHS).

**Methods:**

This study was designed as an open prospective individual randomized controlled trial. Eligible participants were aged 35 to 65 years with a body mass index (BMI) of ≥25.0 in their latest health examination. Participants were randomly assigned to either the WSHS group or the EHS group. Thirteen registered dietitians under the direction of a principal dietitian each instructed 6 to 8 participants from the respective groups. All participants in the WSHS group could receive nutritional advice and calculate their nutritive intake from a photograph of a meal on their computer screen from the Internet sent to them by their dietitian, receive supervision from the registered dietitian, and view fellow participants’ weight changes and lifestyle modifications. In the EHS group, a participant could receive one-on-one nutritional advice and calculate his/her nutritive intake from the photograph of a meal on computer screen sent by email from his/her dietitian, without being able to view fellow participants’ status. The follow-up period was 12 weeks for both groups. The primary outcome measure was change in body weight. The secondary outcome measure included changes in BMI and waist circumference. The intergroup comparison of the changes before and after intervention was evaluated using analysis of covariance.

**Results:**

A total of 193 participants were randomly assigned to either the WSHS group (n=97) or the EHS group (n=96). Ten from the WSHS group and 8 from the EHS group dropped out during the study period, and the remaining 87 in the WSHS group and 88 in the EHS group were followed up completely. Weight loss was significantly greater in the WSHS group than in the EHS group (–1.6 kg vs –0.7 kg; adjusted *P*=.04). However, there were few differences in waist circumference between the 2 groups. (–3.3 cm vs –3.0 cm; adjusted *P*=.71).

**Conclusions:**

Our newly developed WSHS system using forced self-disclosure had better short-term weight loss results. Further study in a longer-term trial is necessary to determine what effects this type of intervention might have on long-term cardiovascular disease.

**Trial Registration:**

University Hospital Medical Information Network Clinical Trial Registration (UMIN-CTR): UMIN000009147; https://upload.umin.ac.jp/cgi-open-bin/ctr/ctr.cgi?function=brows&action=brows&type=summary&recptno=R000010719&language=E (Archived by WebCite at http://www.webcitation.org/6HTCkhb1p).

## Introduction

Obesity is one of the most common public health problems in the industrialized world as a cause of noncommunicable diseases, such as ischemic heart disease and diabetes mellitus [1,2]. It has been reported that people are more likely to gain weight when obese persons are around them [3]. In a similar way, behavior modification for weight loss might also transmit to others if a person makes an effort to lose weight. In addition, the necessity to enhance the motivation for weight loss in nutritional counseling has been emphasized [4].

In nutritional counseling for weight loss, face-to-face support that takes into consideration the individual’s background and personal characteristics is generally conducted by registered dietitians [5,6]. Recently, emails, which are primarily for one-on-one communication, have been used for weight loss [7-12]. Self-disclosure plays a central role in the development and maintenance of relationships [13], and is also thought to be a critical component in enabling the therapeutic progress [14]. Although writing about experiences of weight loss through blogging as a means of self-disclosure has expanded rapidly recently [15], it is unclear whether forced self-disclosure via the Internet would be actually effective for weight loss.

We developed a Web-based self-disclosure health support (WSHS) system through which participants can receive counseling from a registered dietitian and compare their own changes in weight and lifestyle with those of others. This study aimed to compare the weight loss between the WSHS and the email health support (EHS). Our hypothesis is that weight loss would be greater in the WSHS group than in the EHS group.

## Methods

### Study Design

This study was an open prospective individual randomized controlled trial (UMIN000009147), carried out from July 2008 through February 2009.

### Study Participants

For this study, we recruited participants by mail, contacting clients of the Kyoto University Health Service, Japan, urging them to obtain nutritional counseling for weight loss. Men and women aged 35 to 65 years with a body mass index (BMI) of 25.0 kg/m^2^ or more from their latest health examination were eligible. Persons who agreed to participate in our study were invited to an initial face-to-face guidance interview. At this interview, those who had been receiving dietary and exercise therapies, or who could not access Internet or email, or who had a current BMI less than 24.5 kg/m^2^ were excluded from our intervention.

### Baseline Measurements

At the first guidance interview, we obtained written informed consent and baseline characteristics, such as sex, age, body height and weight, and waist circumference, and established the participants’ own target level of weight loss. In addition, we conducted a baseline questionnaire survey on the participants’ quality of life (QOL) [16,17]. QOL was measured using the Medical Outcomes Study Short-Form 36 survey (SF-36) [16,17], which is a self-reported measure that assesses 4 separate QOL domains, including general health perception, vitality, role of functioning related to physical and emotional problems, and mental health. Higher scores indicate a more positive health-related QOL for each item [16,17].

### Randomization

The participants were randomly assigned to either the WSHS group or the EHS group using the minimization method, balancing sex (male or female), age (<40 years or ≥40 years), and baseline body weight (<60 kg, 60-80 kg, or ≥80 kg) by 1 of the authors (MA). Then they were assigned a counselor-dietitian. A total of 13 registered dietitians under the direction of a principal dietitian provided nutritional counseling. Each dietitian was allocated to both 1 of the WSHS groups and 1 of the EHS groups to minimize the intergroup differences in dietitians’ counseling, with 6 to 8 participants of a group being supported by 1 assigned dietitian during the study period. Each dietitian uniformly counseled the participants in both groups based on the standardized manual on the nutritional values of diet records and a photograph of a meal [18] provided by the principal dietitian to maintain the homogeneity of guidance among dietitian counselors. The follow-up period was 12 weeks for both groups.

### Interventions and Follow-up

**Figure 1:**
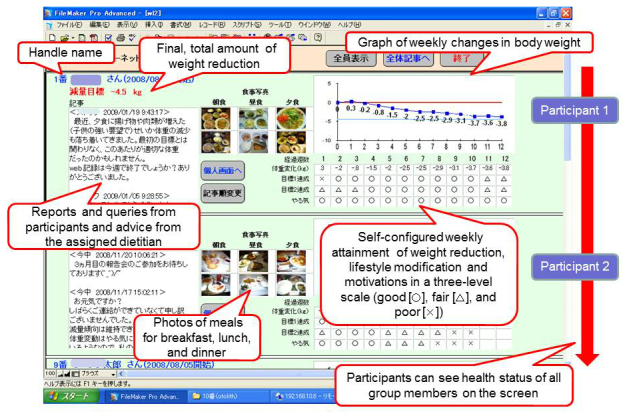
Screen view of the Web-based self-disclosure (WSHS) health support system.

The WSHS group members were given a personal account and password, and could freely access the WSHS system (Figure 1). Each participant set his/her own username and target body weight at the beginning. All members were requested to fill in their present body weight and the level of their lifestyle modification attained such as food records and exercise, along with their motivation level, which were expressed in a 3-level scale (good, fair, and poor), on the screen of the individual’s system Web page every week. Participants received nutritional advice and had their nutritive intakes calculated by their dietitians using a photograph of a meal. A participant and his/her dietitian could discuss their questions and comments in this personal area. In this system, group members could view their fellow participants’ weight changes (not actual values) and their related conditions. A participant and his/her dietitian could put their queries or comments on the participant’s individual screen, but fellow participants could not write in this column.

The EHS group members were provided with a Microsoft Excel file. They set their target body weight loss at the beginning, and subsequently filled in their present body weight, their levels of lifestyle modification attainment, and their motivation level, similar to the WSHS group members. They could send questions and receive nutritional advice and photo-based nutritive intakes by email. However, the EHS was not a Web-based system, and the participants could not obtain information on their fellow participants’ health status via the Web.

The difference between the 2 interventions was that WSHS participants could receive advice from the corresponding dietitian and view other participant’s progress when they accessed this system, and EHS participants could only receive advice from the corresponding dietitian. After 12 weeks of online health support, the participants were asked to come in for remeasurement of their height, weight, waist circumference, and QOL by the same dietitian they saw at the beginning of the study.

### Statistical Analysis

The primary outcome measure was change in body weight. The secondary outcome measure included changes in BMI, waist circumference, and QOL.

The sample size was calculated based on weight loss during the 12 weeks. We hypothesized that participants assigned to the WSHS group would lose a mean of 2.0 kg after the 12-week intervention, compared with a loss of 1.0 kg in the EHS group with standard deviations of 2.0 kg for both groups [19-21]. Based on 0.9 power to detect a significant difference (*P*=.05, 2-sided), 85 participants were required for each study group. To compensate for possible absences, we enrolled 90 participants per group.

We conducted intention-to-treat analyses in this study. All data are expressed as mean (SD). BMI was calculated as weight/height^2^. Baseline characteristics were compared between the groups using unpaired Student *t* test for numerical variables and Pearson chi-square test for categorical variables. The comparison of changes before and after intervention between the groups was evaluated using analysis of covariance adjusted for sex, age, and the baseline value of the corresponding item at the first guidance interview. All statistical analyses were performed using JMP 9 statistical software (SAS Institute, Inc, Cary, NC, USA). All tests were 2-tailed and *P* values of <.05 were considered statistically significant.

### Ethical Considerations

All procedures were conducted according to the Declaration of Helsinki. Participants submitted their written informed consent before participation. This study was approved by the Ethics Committee of Kyoto University Graduate School of Medicine.

## Results

**Figure 2:**
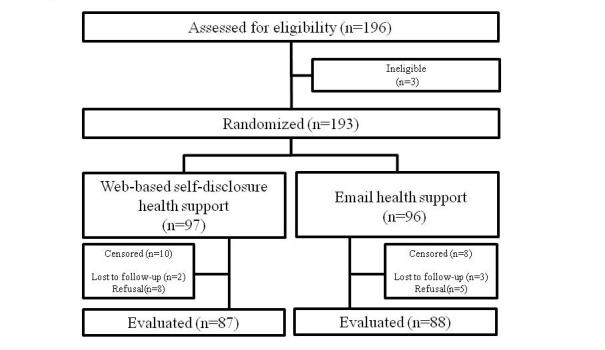
Patient flow.

A total of 196 participants were recruited for this trial from July 2008 through February 2009. Among them, 3 participants with a BMI of <24.5 at the first guidance interview were excluded, and the remaining 193 were randomly assigned to either the WSHS group (97) or the EHS group (96). Ten persons from the WSHS group and 8 from the EHS group dropped out during the study period, leaving 87 in the WSHS group and 88 in the EHS group to complete the study (Figure 2).

Baseline characteristics of the participants are shown in Table 1. Mean age was 50 years (SD 7), and mean body weight was approximately 78 kg (SD 10) in both groups. There were no significant differences in sex ratio, BMI, waist circumference, target body weight loss, or QOL levels between the groups.

Differences in changes of outcomes between the WSHS group and EHS group are shown in Table 2. The loss in body weight was significantly greater in the WSHS group than in the EHS group (–1.6 kg versus –0.7 kg; adjusted *P*=.04). The decrease in BMI tended to be also greater in the WSHS group than in the EHS group (–0.6 versus –0.3; adjusted *P*=.05) although it was statistically insignificant. There were no significantly different changes in waist circumference. Changes in QOL scores, general health perception, vitality, role functioning related to physical and emotional problems, and mental health were not significantly different between the groups.

**Table 1:** Baseline characteristics of participants (N=193).

Participants’ characteristics		Web-based self-disclosure health support (n=97)	Email health support (n=96)	*P* value
**Fundamental characteristics**				
	Male, n (%)	84 (86.6)	82 (85.4)	.81^a^
	Age (years), mean (SD)	50.7 (7.4)	49.6 (7.2)	.31^b^
**Physical characteristics, mean (SD)**				
	Body weight (kg)	78.8 (10.6)	77.1 (9.6)	.23^b^
	Body mass index (kg/m^2^)	27.5 (3.1)	27.4 (2.5)	.28^b^
	Waist circumference (cm)	94.9 (7.2)	93.6 (7.2)	.26^b^
	Target weight loss (kg)	–4.5 (1.7)	–4.5 (1.9)	.95^b^
**Quality of life from SF-36,** ^c^ **mean (SD)**				
	General health perception	52.1 (17.3)	52.7 (13.9)	.79^b^
	Vitality	55.0 (15.9)	55.4 (16.3)	.86^b^
	Role functioning^d^	67.1 (21.3)	69.9 (17.7)	.34^b^
	Mental health	66.0 (17.0)	66.2 (14.6)	.93^b^

^a^Pearson chi-square test.

^b^Student *t* test.

^c^Data from 86 participants in the Web-based self-disclosure health support group and 88 participants in the email health support group.

^d^Role functioning: role functioning related to physical and emotional problems.

**Table 2:** Changes in physique and quality of life before and after intervention between groups (N=175).

Changes in outcomes		Web-based self-disclosure health support (n=87)	Email health support (n=88)	*P* value^a^	
				Unadjusted	Adjusted^b^
**Physical changes mean (SD)**					
	Body weight (kg)	–1.6 (2.7)	–0.7 (2.3)	.02	.04
	Body mass index (kg/m^2^)	–0.6 (1.0)	–0.3 (0.8)	.03	.05
	Waist circumference (cm)	–3.3 (3.3)	–3.0 (3.9)	.64	.71
**Changes in items on quality of life from SF-36,** ^c^ **mean (SD)**				
	General health perception	0.4 (13.3)	–1.4 (11.5)	.44	.36
	Vitality	0.0 (13.3)	–0.6 (13.3)	.80	.98
	Role functioning^d^	4.0 (26.1)	4.7 (19.1)	.89	.70
	Mental health	0.5 (17.1)	1.1 (11.1)	.82	.66

^a^Analysis of covariance.

^b^ *P* values were adjusted for sex, age, and the baseline value of the corresponding item at the first guidance interview.

^c^Data from 57 participants in the Web-based self-disclosure health support system group and 59 participants in the email health support group were acquired.

^d^Role functioning: role functioning related to physical and emotional problems.

## Discussion

### Principal Findings

We developed the WSHS system, which is capable of activating self-disclosure, and evaluated its effectiveness in controlling body weight in a randomized controlled trial. In this trial, the WSHS yielded participants with a significantly greater weight loss than did the EHS. Although previous studies developed and evaluated weight loss applications [22-25], our WSHS system is a first step in exploring the benefits of a system in which there is forced self-disclosure and helpful information on nutritional counseling.

It is well-known that face-to-face counseling is effective for weight loss and blood sugar control [5,6]. However, it would be exceedingly difficult to continue face-to-face support for busy middle-aged workers who are candidates for cardiovascular diseases [8,26]. Here, Internet health care support, including email counseling, has shown itself to be similarly effective as the face-to-face method [7-12]. Because Internet services, including e-learning, are useful [22,23], our WSHS system can also be a useful tool for people who need health support. In addition, the beneficial effects of self-disclosure in health care counseling should be emphasized [4]. The participants were fully aware of their fellow participants’ health status, which might have encouraged them to attain their own health goals.

Although reduction in body weight was significantly greater in the WSHS group than in the EHS group, there were no significant differences in waist circumference between the groups. Intra-abdominal fat accumulation of 100 cm^2^ or more is one of the cardiovascular risk factors [27]. An 85-cm waist circumference corresponds to approximately 100 cm^2^ of intra-abdominal fat accumulation, and is used as a simple diagnostic criterion of metabolic syndrome in Japan [28]. It is known that the measurement of waist circumference varies with participants’ intention to flatten their stomach [29], and this flattening might have affected the evaluation of waist circumference in this study.

This study also evaluated 4 SF-36 items as weight loss-associated QOL, but there were few differences in the changes between groups. In previous studies, QOL in obese persons was lower because they were more likely to have low back pain, joint disorders, sleep disorders, and depression [30-32]. Patients with metabolic syndrome were also more likely to be depressive [33]. Furthermore, persons who had succeeded in weight loss improved their own general perception of health and physical functioning [31,34]. The short length of our study or the small intergroup difference in weight loss might have blurred the effects on QOL. Further investigation is needed to evaluate whether WSHS will lead to greater QOL [35]. The number of Internet users reached approximately 2.3 billion people in 2012 across the globe [36]. Therefore, nutritional counseling via the Internet can reach more people and offer more continuous professional support [37]. The WSHS system might cost more to produce than the EHS system; therefore, the cost-effectiveness is an important issue that should be discussed in the future. However, the WSHS systems using forced self-disclosure could be a promising means for decreasing the current high rates of obesity.

### Limitations

This study has some inherent limitations. First, the WSHS system is limited to those who can use a personal computer. Second, we only observed the effect on weight loss. The true endpoint of nutritional counseling should be the reduction in mortality and morbidity from cardiovascular diseases. Weight loss could prevent cardiovascular events [38]. Therefore, we consider that weight change is reasonable as a short-term index. Third, lack of information on the number of interactions over the computer/Web interface over the study period and the dietitian interaction with each participant was another limitation because their differences might influence the difference in weight loss among participants. Fourth, information was lacking on social influence on participants’ behaviors, which might also have a possible effect on the change of weight loss. Finally, although weight change has been commonly used as a reasonable short-term index, the short observation period of this study is another important limitation because obesity is a chronic condition and requires long-term solutions.

### Conclusions

Our newly developed WSHS system using forced self-disclosure would be significantly more effective than the EHS system for short-term weight loss. A longer-term trial that further explores the theoretical differences between these 2 interventions would be necessary to draw conclusions about the WSHS effect on longer-term health conditions.

## Multimedia Appendix 1

CONSORT-EHEALTH checklist V1.6.2 [39].

## Abbreviations

BMI: body mass index
EHS: email health support
QOL: quality of life
WSHS: Web-based self-disclosure health support
